# Selection of Osteoprogenitors from the Jaw Periosteum by a Specific Animal-Free Culture Medium

**DOI:** 10.1371/journal.pone.0081674

**Published:** 2013-12-09

**Authors:** Dorothea Alexander, Melanie Rieger, Christian Klein, Nina Ardjomandi, Siegmar Reinert

**Affiliations:** 1 Department of Oral and Maxillofacial Surgery, University Hospital of Tübingen, Tübingen, Germany; 2 Department of Conservative Dentistry, University Hospital of Tübingen, Tübingen, Germany; 3 Dental practice Zahngesundheit Waiblingen, Waiblingen, Germany; National Institutes of Health, United States of America

## Abstract

The goal of our research work is to establish mesenchymal osteoprogenitors derived from human jaw periosteum for tissue engineering applications in oral and maxillofacial surgery. For future autologous and/or allogeneic transplantations, some issues must be addressed. On the one hand, animal-free culture conditions have yet to be established. On the other hand, attempts should be undertaken to shorten the *in vitro* culturing process efficiently. The aim of the present study is to compare and analyze the phenotype of osteoprogenitors from the jaw periosteum under normal FCS-containing and animal-free culture conditions. Therefore, we analyzed the proliferation rates of MesenCult-XF medium (MC-) in comparison to DMEM-cultured JPCs. Whereas jaw periosteal cells (JPCs) show relatively slow proliferation rates and a fibroblastoid shape under DMEM culture conditions, MC-cultured JPCs diminished their cell size significantly and proliferated rapidly. By live-monitoring measurements of adhesion and proliferation, we made an interesting observation: whereas the proliferation of the MSCA-1^+^ subpopulation and the unseparated cell fraction were favored by the animal-free culture medium, the proliferation of the MSCA-1^-^ subpopulation seemed to be repressed under these conditions. The alkaline phosphatase expression pattern showed similar results under both culture conditions. Comparison of the mineralization capacity revealed an earlier formation of calcium-phosphate precipitates under MC culture conditions; however, the mineralization capacity of the DMEM-cultured cells seemed to be higher. We conclude that the tested animal-free medium is suitable for the *in vitro* expansion and even for the specific selection of osteoprogenitor cells derived from the jaw periosteum.

## Introduction

In previous studies, we characterized the phenotype, osteogenic potential and features of jaw periosteal cells (JPCs) grown in two- and three-dimensional culture conditions [1]–[7]. Unfortunately, we also determined that not all isolated jaw periosteal cells are able to mineralize *in vitro*. Due to this fact, the identification of specific markers for the osteogenic progenitors from the jaw periosteum is of paramount importance for the selection of these cells from the whole heterogeneous cell population. Screenings for cell surface markers revealed that two mesenchymal stem cell markers were significantly induced during the osteogenesis of jaw periosteal cells. We were able to show that the stem cell marker CD271 (LNGFR – low affinity nerve growth factor receptor) was significantly more induced in mineralizing than in non-mineralizing periosteal cells during osteogenic induction. However, we demonstrated that this antigen does not designate the subpopulation with the highest osteogenic potential. In contrast, the cell surface antigen mesenchymal stem cell antigen-1 (MSCA-1) was shown to be significantly more induced in mineralizing compared to non-mineralizing jaw periosteal cells, both in the undifferentiated and differentiated states. Additionally, we could prove that the MSCA-1-positive subpopulation exhibits a significant higher osteogenic capacity than the respective MSCA-1-negative subpopulation; therefore, it most likely represents the osteoprogenitor fraction from the entire cell population [3]. Being an extremely rare population, a long *in vitro* expansion of the entire population is required before magnetic separation of the MSCA-1+ subpopulation can be performed. However, the long *in vitro* passaging of the cells is accompanied by undesirable increases in the occurrence of cellular senescence. Therefore, the shortening of the *in vitro* expansion procedure, as well as the use of serum-free culture conditions, should be achieved to ensure the success of future tissue engineering applications using cell-based constructs.

We focused the present study on the comparison of the JPC phenotype under FCS-containing and FCS-free culture conditions and analyzed in detail the proliferation and mineralization capacities of the cells, as well as their expression of common stem cell and osteogenesis-relevant markers.

## Materials and Methods

### Cell culture

Human jaw periosteum biopsies were obtained during routine oral and maxillofacial surgery after obtaining written informed consent. Samples from 6 donors (average age 57, 3 healthy donors with fractures of the midface and 3 donors suffering from squamous cell carcinoma) were included in this study in accordance with the local ethical committee (Ethik-Kommission der Medizinischen Fakultät Tübingen, approval number 194/2008BO2). After breaking down the jaw periosteal tissue followed by a main digestion step using type XI collagenase (1500 U/ml, Sigma-Aldrich, Steinheim, Germany) for 90 min, the JPCs were plated into 75 cm^2^ culture flasks. For JPC expansion under FCS-containing and animal–free culture conditions, cells were cultured in DMEM/F-12 (Invitrogen-BioSource Europe, Nivelles, Belgium) containing 10% FCS (Sigma-Aldrich, Steinheim, Germany) and 1% fungicide and penicillin/streptomycin (Biochrom, Berlin, Germany) and/or in MesenCult-XF medium (MC-XF - Stemcell Technologies, Grenoble, France) containing 1% glutamine and 1% fungicide and penicillin/streptomycin. DMEM-cultured cells were passaged using trypsin-versene EDTA (1×, Lonza, Basel, Schweiz), and MC-XF-cultured cells were passaged using the MesenCult-ACF dissociation kit. Furthermore, for MC-XF culture conditions, culture dishes or flasks were coated overnight with MesenCult-XF attachment substrate provided from the same company.

For the calculation of the population doubling times, the individual constant for each fraction/patient was taken into consideration and firstly determined according the following formula:



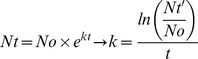



whereby: Nt =  cell number (as counted using the Neubauer counting chamber) at time t; No =  cell number at time 0; k =  constant; t =  number of days in culture, were included in the calculation.

On the basis of an expected exponential cell growth, the population doubling time (t′) was calculated according following formula:
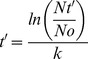
whereby: Nt′ = 2×No and t′ = ln2/k, is.

The obtained values (n = 3 or n = 4, as indicated in the table) for the population doubling times are summarized in table 1.

**Table 1:** PDT (days ± STD) during in vitro passaging.

Passage	DMEM	MC-XF	Sample size	p-value
4	6.82±3.03	3.65±1.12	4	n.s. (<0.07)
5	5.41±2.80	2.29±0.50	4	n.s. (<0.06)
6	3.67±0.31	2.12±0.08	3	<0.01
7	3.32±0.53	2.44±0.76	3	<0.05

Overview of calculated average population doubling times (PDT, in days, ± STD) in JPCs cultured under DMEM and MC-XF conditions (n = 3 or n = 4, as indicated in the table) at different passages (passage 4–7).

### Differentiation experiments

DMEM-cultured JPCs (4×10^4^ cells per well in 6-well plates) were treated with osteogenic medium (ob - DMEM/F12 containing 10% FCS, 10 mM β-glycerophosphate, 100 µM L-ascorbic acid 2-phosphate and 4 µm dexamethasone, Sigma-Aldrich) for 30 days. The medium was replaced three times per week. Untreated cells that were cultivated without any osteogenic compounds for the same period served as undifferentiated controls (co).

Additionally, 6×10^4^ MC-XF-cultured JPCs were plated into 6-well plates that had been coated with MC-XF attachment substrate for osteogenic stimulation using the MesenCult osteogenic medium (ob - containing MesenCult MSC basal medium, 5% osteogenic stimulatory supplement and 3.5 mM β-glycerophosphate) for the same time period as DMEM-cultured cells. In this case as well, untreated MC-XF-cultured JPCs served as undifferentiated controls (co).

### Life-monitoring of cell proliferation using the xCELLigence system

The xCELLigence system (ACEA Biosciences, San Diego, U.S.A.) is suitable for the real-time analysis of cell proliferation, migration and invasion. Measurements are based on electric cell impedance-dependent signals (micro-electrodes are integrated onto the bottoms of culture E-plates) due to cell adherence and spreading for label-free cell analysis. The cell index (CI) was derived to represent cell status based on the measured electric cell impedance.

For life-monitoring measurements, 2×10^3^ cells (of passage 4–5) were plated per well into uncoated special E-plates with a total volume of 200 µl per well.

**Figure 1:**
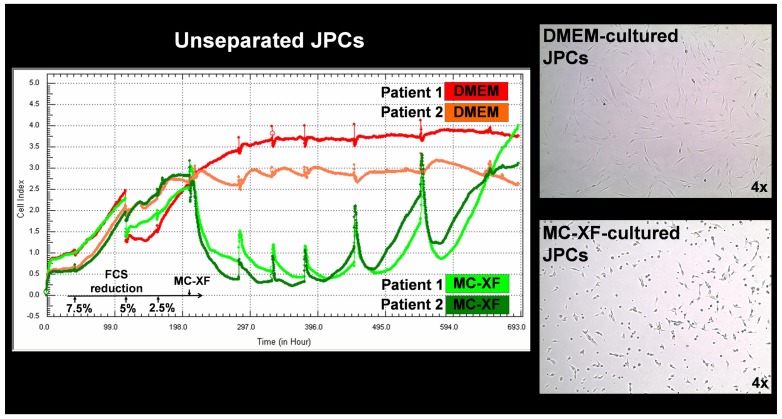
Life-monitoring measurements of cell proliferation by unseparated JPCs using the x-CELLigence system (ACEA Biosciences). JPCs of passage 4 derived from two different donors were seeded into special E-plates in DMEM/F12/10%FCS culture medium. Two days later (44 hours - each tick of the scale corresponds to 11 hours), a gradual FCS reduction was performed in one-half of the test runs (green and dark green), whereas the other half of the wells was further cultivated in DMEM/F12/10%FCS (red and coral). Nine days (297 hours) after the initiation of FCS reduction, MC-XF culture medium was added to the cells. The proliferation curve progression of DMEM-cultured JPCs (from two representative patients) is highlighted in red and coral and that of MC-XF-cultivated cells is highlighted in dark green and green. The right panel of the figure shows the cell morphology of DMEM- and MC-XF-cultured JPCs. Note the reduced cell size under MC-XF culture conditions (on uncoated dishes) leading to the significant decrease of cell impedance immediately after the addition of the MC-XF culture medium.

**Figure 2:**
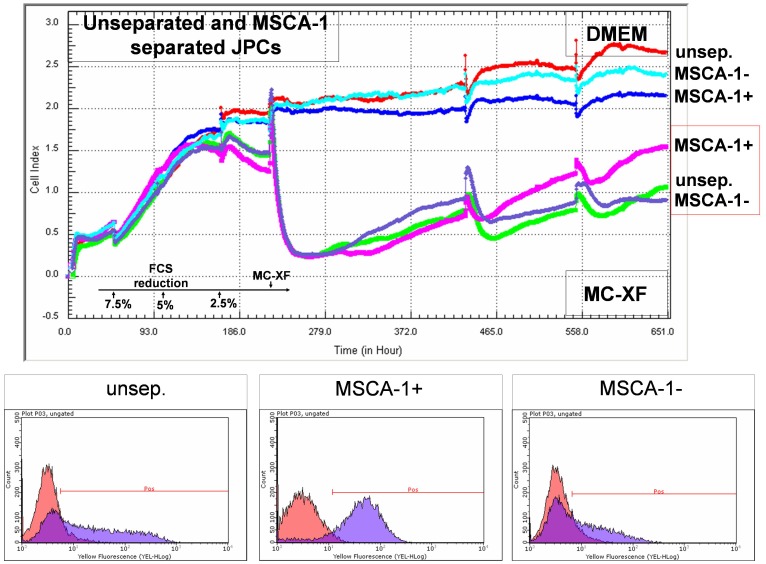
Life-monitoring measurements of cell proliferation by unseparated and MSCA-1-separated JPCs using the x-CELLigence system (representative curve progression of cells derived from one donor). Cells (of passage 5) were seeded into E-plates for the life-measurements of cell impedance following the same experimental procedures as described in fig. 1 (gradual FCS reduction and addition of the MC-XF culture medium). Note the preferential proliferation activation of the MSCA-1^+^ subpopulation (pink line) in contrast to the rather restrictive capacity of the xenogeneic-free culture medium on MSCA-1^−^ JPCs (violet line). The lower panel of the figure illustrates representative histograms of MSCA-1 expression by flow cytometry before (unsep.) or after the magnetic separation of the MSCA-1+/− fractions.

For the comparison of cell proliferation under both media conditions, cells were first plated in DMEM/F-12 containing 10% FCS. Approximately 2 days later (44 hours in fig. 1 and 55 hours in fig. 2), a gradual serum reduction was started (7.5, 5, 2.5%). Three days (65 hours) after the addition of DMEM containing 2.5% FCS, MC-XF medium was added to the cell cultures. As controls for the comparative analyses, cells derived from the same patient were cultured in DMEM/F-12 (10% FCS) in parallel experiments.

### Flow cytometry analysis of mesenchymal stem cell marker and MSCA-1 expression

Unseparated JPCs were detached from culture dishes, centrifuged (350 x*g*, 7 min), resuspended in 20 µl of 10% Gamunex (human immune globulin solution, Talecris Biotherapeutics, Frankfurt, Germany) and incubated for 15 min at 4°C. After adding 100 µl of FACS buffer (PBS, 0.1% BSA, 0.1% sodium azide), the cells were incubated with specific phycoerythrin (PE)-labeled mouse anti-human CD29, CD45, CD73, CD90 (BD Biosciences Pharmingen, San Diego, U.S.A.), CD105 (AbD Serotec) and MSCA-1 (MACS Miltenyi Biotec, Bergisch Gladbach, Germany) for 15 min at 4°C. The cells were centrifuged (350 x*g*, 7 min) and washed two times with FACS buffer and resuspended in 200 µl of FACS buffer for flow cytometric analysis. The measurements were carried out with the guava easyCyte 6–2 L flow cytometer (Merck Millipore, Darmstadt, Germany). Cells incubated with polyclonal goat anti-mouse immunoglobulins served as negative controls. For data evaluation, guavaSoft 2.2.3 (InCyte 2.2.2) software was used.

### Detection of cell mineralization by fluorescent OsteoImage staining

We detected *in vitro* JPC mineralization by Alizarin or von Kossa staining. Based on the fact that the specificity for hydroxyapatite of both stains has been controversially discussed [8], [9], we used the fluorescent OsteoImage mineralization assay, which promised to be more specific for inorganic hydroxyapatite. Therefore, undifferentiated and osteogenic-induced JPCs cultured under both media conditions were fixed with zinc-formaline for 20 min. After three washing steps, cells were incubated with diluted staining reagent in the dark for 30 min, following the manufacturer's instructions. After washing, the stained cells were visualized using the Observer.Z1 microscope with the apotome technique (Zeiss).

### Gene expression analysis in DMEM- and MC-XF-cultured JPCs by quantitative PCR

RNA isolation from DMEM- and MC-XF-cultured unseparated JPCs (n = 4 for each group) was carried out using the NucleoSpin RNA XS kit (Macherey-Nagel, Düren, Germany) following the manufacturer's instructions. The amount of isolated RNA was photometrically measured and quantified (GeneQuant Pro; GE Healthcare) and 15 ng of RNA was used for cDNA synthesis using the QuantiTect Whole Transcriptome Kit (Qiagen, Venlo, Holland) following the manufacturer's instructions.

The quantification of mRNA expression levels was performed using the real-time LightCycler System (Roche Diagnostics, Mannheim, Germany). For the PCR reactions, commercial primer kits (Search LC, Heidelberg, Germany) and DNA Master Sybr Green 1 (Roche) were used. The amplification was performed with 35 cycles. The transcript levels were normalized to those of the housekeeping gene glycerinaldehyde 3-phosphate dehydrogenase (GAPDH), and the induction factors of the osteogenic induced samples in relation to those of untreated samples were calculated.

### Statistical analysis

The data are expressed as the mean ± standard deviation. For the statistical analysis, Student's t-test was used. A p-value <0.05 was considered significant.

## Results

### *In vitro* cultivation of JPCs under FCS-containing and FCS-free culture conditions

For the explicit comparison of both culture conditions (DMEM/F-12 and MC-XF), cell morphology was first analyzed by light microscopy. Compared to DMEM-cultured JPCs that showed an elongate and fibroblastoid shape, MC-XF-cultured cells significantly diminished their cell size. Additionally, we detected an earlier formation of calcium phosphate precipitates in MC-XF-cultured JPCs at day 12 of osteogenesis. Furthermore, the cell population seemed to be more homogenous, due to appearance of nodules derived from almost each cell of the entire monolayer. In contrast, the mineral deposition of DMEM-cultured cells originated from individual cells; these mineralization centers increased continuously.

The examination of the population doubling times revealed higher proliferation rates of MC-XF- in comparison to DMEM-cultured JPCs as determined for four passages (passage 4–7). An overview of the calculated population doubling times (PDT) at indicated passages is given in table 1. The PDT of MC-XF-cultured JPCs of passage 6 and 7 (n = 3) were shown to be significant lower than those obtained by DMEM- cultivated cells indicating the faster proliferation capacity of MC-XF-cultivated cells. The same tendency combined with much higher differences (PDT of DMEM-cultured cells was shown to be on average 3 days longer than those of MC-XF-cultured JPCs) were obtained at passage 4 and 5 (n = 4) however, differences did not reach significance.

### Life-monitoring of cell proliferation using the xCELLigence system

Cell proliferation was analyzed by life-monitoring measurements using the xCELLigence system (ACEA Biosciences). In the first step, the cell impedance of unseparated JPCs derived from 2 patients was analyzed, as shown in fig. 1. Interestingly, serial FCS reduction did not significantly impair cell proliferation. After the addition of MC-XF, cell impedance decreased significantly, and cell proliferation recovered after approximately 11 days (282 hours). Approximately 18 days (429 hours) after the MC-XF addition, a cross-over of the proliferation curves derived from patient 2 (coral and dark green curve) and from patient 1 (red and green curve) became visible (approx. 20 d or 473 hours).

In successive experiments, unseparated and MSCA-1-separated JPCs were life-monitored using the same system (fig. 2). Serial serum reductions up to 5% did not impair cell proliferation, but a medium change containing 2.5% FCS showed a decrease in the proliferation rates of the separated cell fractions. After the addition of MC-XF, cell impedance decreased significantly. Twenty days after the initiation of the experiment or 10 days (246 hours) after the addition of the MC-XF medium, clear differences between the proliferation curves became apparent. On the one hand, the cell proliferation of the MSCA-1^positive^ fraction was favored, whereas the proliferation of the MSCA-1^negative^ fraction was rather abolished. We reproduced these results in 3 independent experiments with MSCA-1-separated cells derived from 3 different donors.

### Flow cytometry analysis of MSCA-1 expression in DMEM- and MC-XF-cultured JPCs

**Figure 3:**
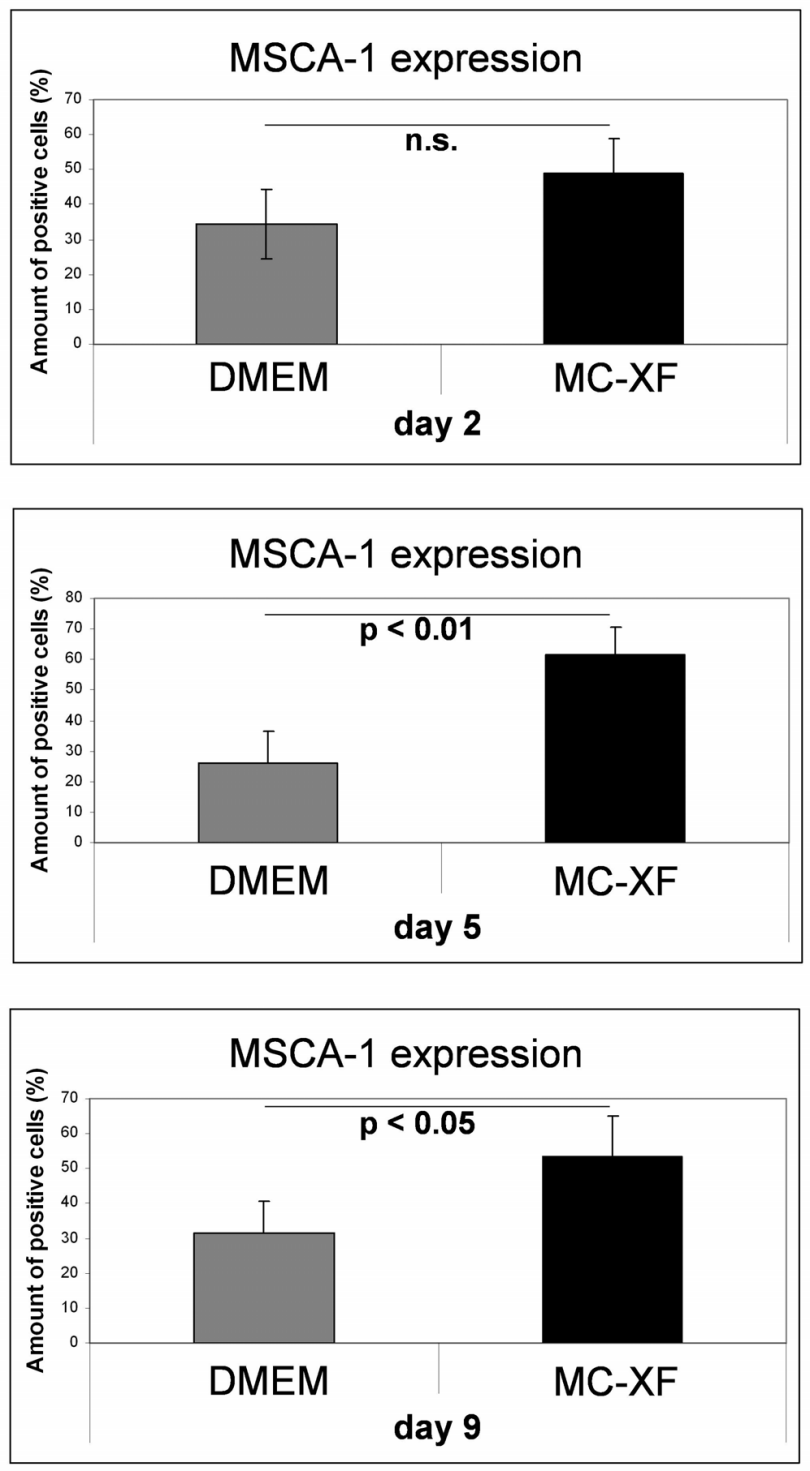
Quantification of MSCA-1^+^ cells under DMEM- and MC-XF culturing conditions by FACS analysis. Unseparated JPCs were plated in culture dishes in DMEM medium. For the first test runs, cells were maintained in DMEM medium whereas the second series underwent stepwise FCS reduction and convertion to MC-XF medium. At the same time points (day 2, 5 and 9 after conversion from DMEM to MC-XF culture conditions), cells were detached from the dishes and the percentages of MSCA-1^+^ cells were determined by FACS analysis. Significant higher amounts of MSCA-1^+^ cells were detected under MC-XF culturing conditions at day 2 and 5 (p<0.01 and p<0.05).

In our experience, the selection of the MSCA-1+ cells takes place within the first 10 days after FCS reduction and subsequent conversion from DMEM to MC-XF medium. For the exact analysis of the percentages of MSCA-1^+^ cells, we performed the experiments exactly the same as for the life-monitoring measurements by xCELLigence and plated 4×10^4^ cells (passage 4–6, n = 5) per well of an uncoated 6-well plate in DMEM medium. Afterwards, parallel experiments were conducted. For the first series, cells were maintained in DMEM medium, whereas the second series underwent stepwise FCS reduction and convertion to MC-XF medium. At the same time points (at day 2, 5 and 9 after conversion of MC-XF to DMEM medium), cells were detached from the dishes and the percentages of MSCA-1^+^ cells were determined by FACS analysis as shown in fig. 3.

We obtained significant higher percentages of MSCA-1+ cells under MC-XF in comparison to DMEM culturing conditions at day 5 (DMEM: 25.82±10.48%; MC-XF: 61.34±9.11%, p<0.01) and at day 9 (DMEM: 31.52±9.14%; MC-XF: 53.34±11.63%, p<0.05) after conversion to MC-XF medium. The same tendency was shown at day 2 without reaching significant values (DMEM: 34.36±10.66%; MC-XF: 48.7±9.85%). Therefore, maximal cell selection with significant higher (on average 35.5%) percentages of MSCA-1^+^ cells was detected at day 5 under MC-XF culture conditions. An overview of the percentages of MSCA-1+ cells under both culture conditions is given in table 2.

**Table 2:** MSCA-1 expression (% positive cells ± STD).

Day	DMEM	MC-XF	Sample size	p-value
2	34.36±10.66	48.70±9.85	5	n.s.
5	25.82±10.48	61.34±9.11	5	<0.01
9	31.52±9.14	53.34±11.63	5	<0.05

Overview of average percentages (± STD) of MSCA-1^+^ cells under DMEM and MC-XF culture conditions (n  =  5) as detected by FACS analysis. Flow cytometric analyses were carried out at day 2, 5 and 9 immediately after conversion from DMEM to MC-XF culture conditions.

### Flow cytometric analysis of mesenchymal stem cell marker expression in DMEM- and MC-XF-cultured JPCs

**Figure 4:**
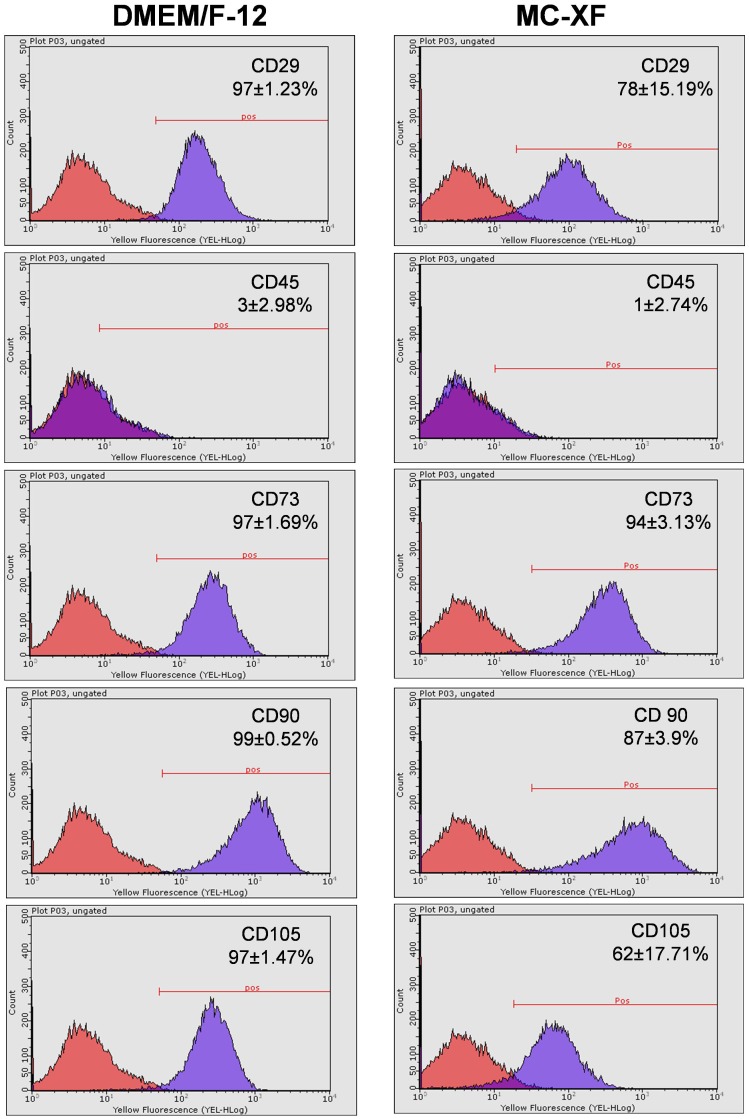
Expression patterns of DMEM- and MC-XF-cultured JPCs (growing within coated flasks) by flow cytometric analyses. Representative histograms and the average percentages (±STD) of positive cells for CD29, CD45, CD73, CD90 and CD105 expression by unseparated JPCs are illustrated.

Cells of passages 5–6 were plated in 75 cm^2^ culture flasks and cultured under both medium conditions for flow cytometric analysis. After reaching 80–90% confluence, the cells were detached from the culture flasks and labeled with specific PE-conjugated antibodies against human CD29, CD73, CD90, CD105, CD166 and CD45. As illustrated in fig. 4, DMEM- and MC-XF-cultured JPCs showed similar expression patterns. They were positive for CD29, CD73, CD90 and CD105 and negative for CD45. MC-XF-cultured cells revealed slightly lower CD29 expression levels (78±15.19 versus 97±1.23, n.s.) however, the obtained values did not reach significance. Significant lower expression of the surface markers CD90 (87±3.90 versus 99±0.52, p<0.05) and CD105 (62±17.71 versus 97±1.47, p<0.05) were detected in these cells in comparison to DMEM-cultured JPCs.

### Analysis of cell mineralization in DMEM- and MC-XF-treated JPCs

**Figure 5:**
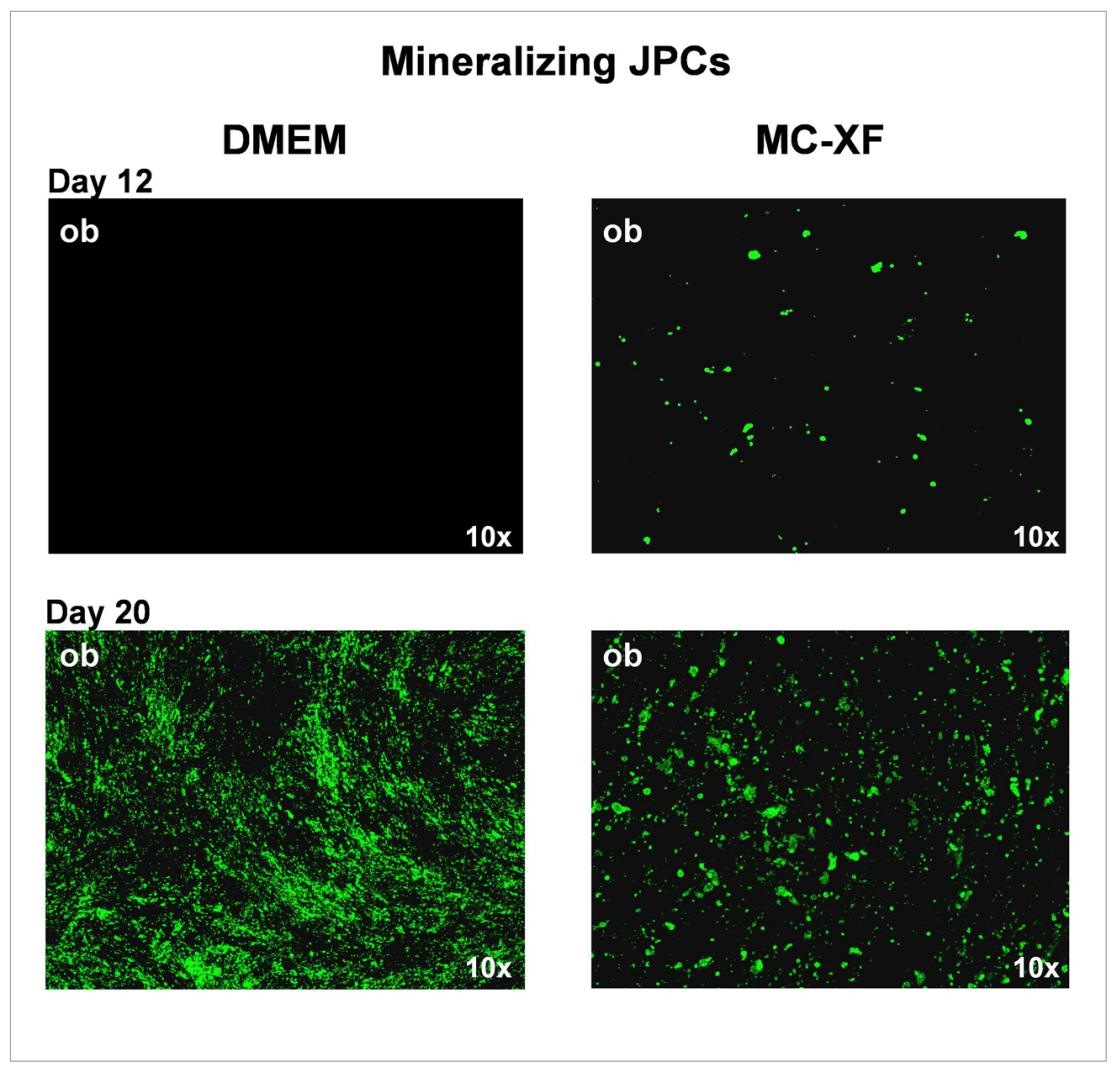
Detection of mineral deposition by mineralizing JPCs (of passage 6). The upper panel of the figure illustrates the beginning of mineralization at day 12 of osteogenic induction (ob) under both media conditions. Note that precipitate formation originates from only a few cells of the DMEM-cultured monolayer. In contrast, the MC-XF-cultivated monolayer seemed to be purer due to the appearance of mineralization potential originating from almost every cell of the monolayer. 4× magnification. The lower panel of the figure illustrates representative fluorescent stainings of hydroxyapatite formation by OsteoImage in DMEM- and MC-XF-cultured monolayers (growing within coated flasks) at day 20 of osteogenesis. JPCs cultivated under DMEM media conditions showed a stronger mineralization potential. 10× magnification.

We observed that the mineralization in MC-XF-cultured mineralizing JPCs (of passage 5–6) occurs earlier (as shown in the upper panel of fig. 5 at day 12 of osteogenesis) than in DMEM-treated cells and originates from almost all cells of the monolayer. However, once precipitate formation was initiated, the hydroxyapatite crystals did not grow to a large extent. In contrast, DMEM-cultivated JPCs showed a later mineralization capacity that emerged only from a few specific cells of the monolayer. The formed precipitates served as a core aggregation point for further crystal deposition. Despite the late start of nodule formation by DMEM-treated cells, specific hydroxyapatite detection by OsteoImage fluorescent staining (fig. 5, lower panel) revealed a higher mineralization capacity of these cells in comparison to MC-XF-cultured JPCs at day 20 of osteogenesis.

**Figure 6:**
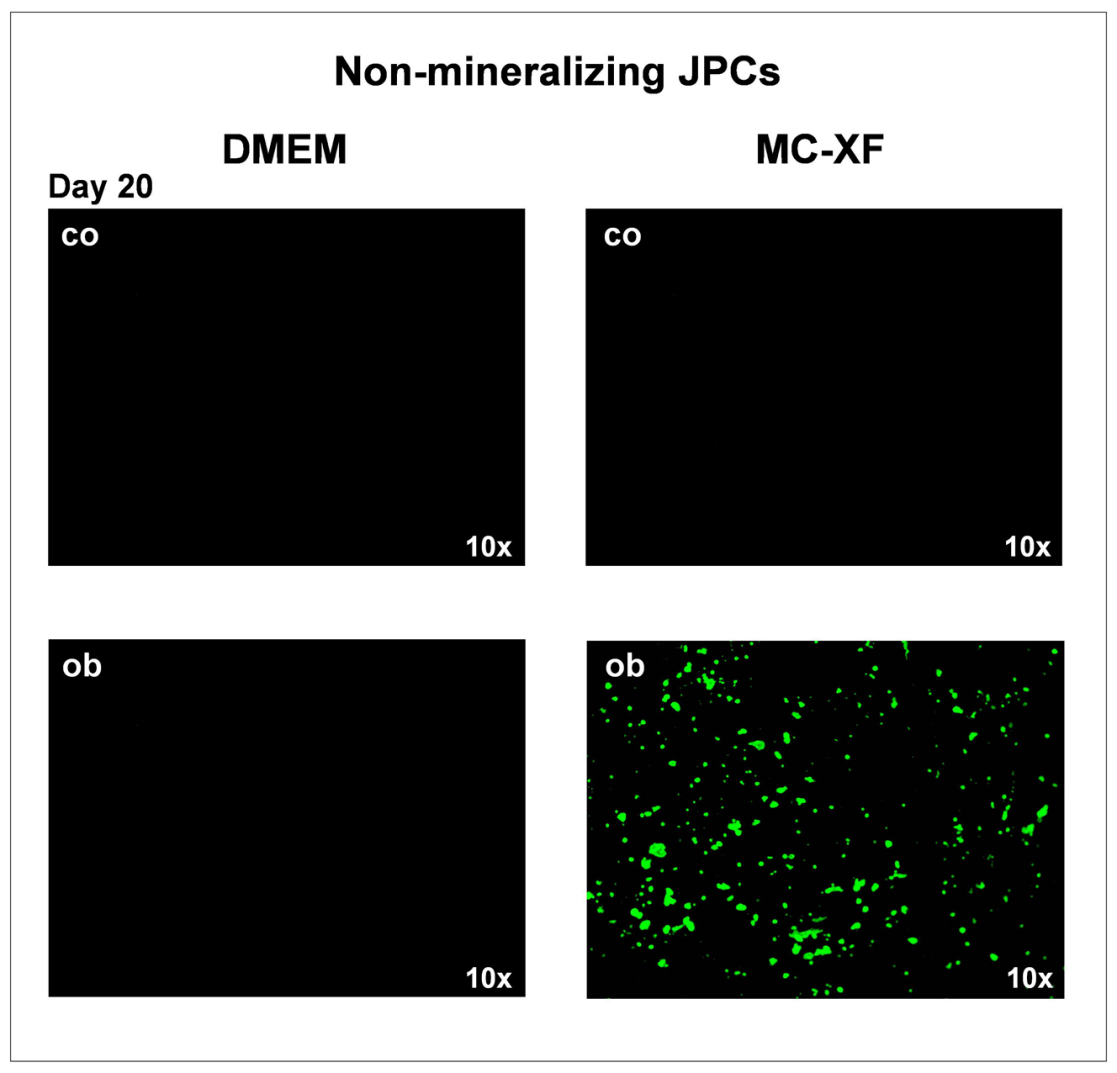
Detection of mineral deposition by non-mineralizing JPCs (of passage 6). Representative fluorescent staining of hydroxyapatite formation by OsteoImage in DMEM- and MC-XF-cultured monolayers (growing within coated flasks) at day 20 of osteogenesis. Non-mineralizing JPCs showed mineralization capacity only under MC-XF but not DMEM media conditions. 10× magnification. Co =  untreated cell, ob =  osteogenic induced cells.

The second particularly interesting observation was that JPCs which were not able to mineralize *in vitro* (non-mineralizing JPCs) under DMEM culture conditions, instead showing mineralization potential under MC-XF cultivation. Representative fluorescent stainings are shown in fig. 6. On the left, the negative staining of non-mineralizing JPCs under DMEM culture conditions in comparison to the positive staining of non-mineralizing JPCs under MC-XF cultivation (right side) is illustrated.

### Gene expression analysis in DMEM- and MC-XF-cultured JPCs

**Figure 7:**
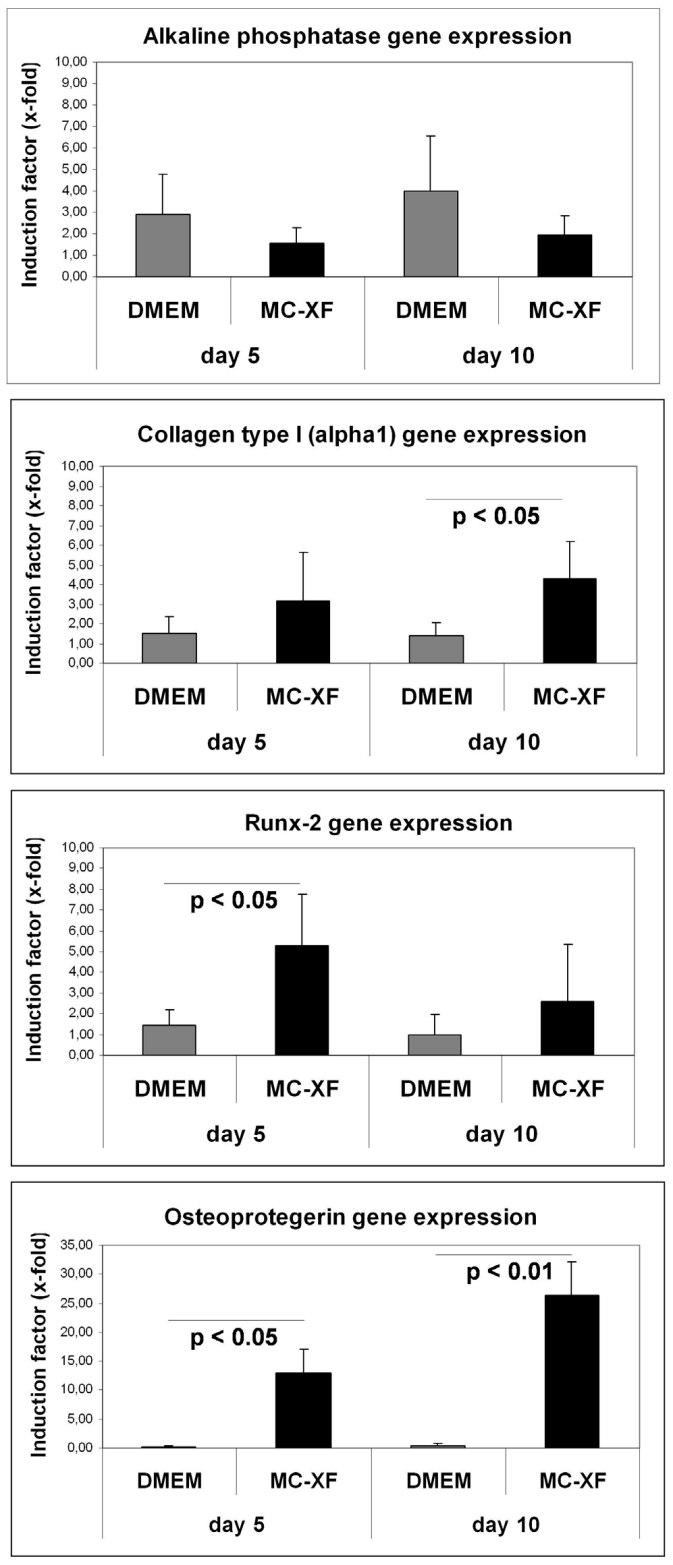
Quantitative analysis of gene expression levels in DMEM- and MC-XF-cultured JPCs at day 5 and 10 of osteogenesis (of passage 6, n = 4). Induction indices (x-fold) and significance values of alkaline phosphatase, Runx-2, type I collagen (alpha1-chain) and osteoprotegerin in osteogenic induced in comparison to untreated cells under both culture conditions are illustrated.

Analysis of the expression of different osteogenesis relevant genes showed only slight differences between DMEM- and MC-XF-cultured unseparated JPCs at day 5 and 10 of osteogenic differentiation (fig. 7). Analysis of the alkaline phosphatase gene expression tends to result in higher mRNA levels in DMEM- compared to MC-XF-cultured JPCs, but the obtained differences in expression did not reach significance. Regarding the gene expression of the alpha1-chain of type I collagen, significantly higher induction levels were detected in MC-XF-cultivated JPCs (4.28±1.91-fold versus 1.37±0.69-fold in DMEM-cultured cells, p<0.05) at day 10 of osteogenesis. Runx-2 gene expression levels were significantly elevated only at the beginning of differentiation (at day 5: 5.31±2.47-fold versus 1.44±0.72 in DMEM-cultured cells, p<0.05). The basal levels of osteoprotegerin were relatively low in MC-XF- compared to DMEM-cultured JPCs, but they were highly inducible during osteogenic differentiation at both analysed time points (12.88±4.11-fold vs. 0.16±0.28-fold (p<0.05) at day 5; 26.45±5.58-fold vs. 0.36±0.42-fold (p<0.01) at day 10). Gene expression analysis of osteocalcin and osteopontin resulted in no significant differences between both cell groups (data not shown).

## Discussion

The treatment of congenital or acquired bone defects is an aspect of major importance in oral and maxillofacial surgery and often subject to significant morbidity following free or microvascular tissue transfer. The aim of our research work is to help patients using bone regenerative techniques. For this purpose, we decided to work with jaw periosteal cells based on the fact that we are convinced that the stem cell niche has an important influence in cell fate determination and genomic imprinting of these stem cells [10] [11]. Furthermore, we prefer to work with this specific cell type due to their localization which commonly overlaps with the site of oral or maxillofacial surgeries to avoid a second intervention.

An important and necessary step in the context of transplantation would be the shortening of the *in vitro* cell expansion procedure. Additionally, with regard to possible disease transmissions or immunological reactions towards xenogeneic compounds, clinical applications should be conducted using mesenchymal stem cells cultured under standardized xenogeneic-free conditions. The immunosuppressive potential of MSCs is well-documented [12]–[20]. However, the use of xenogenic compounds for the expansion and differentiation of these cells could negatively affect these features [21]–[23].

Sotiropoulou and co-authors examined in an extensive study the comparison of different media and plating densities on the phenotype of bone marrow mesenchymal stem cells (BMMSCs). However, the tested media were all supplemented with 10% FCS [24].

For the large production of clinical grade mesenchymal stem cells, the establishment of cell culture methods using chemically defined materials need to be developed [25]. A recent comparative study partially funded by Becton Dickinson describe the growth and differentiation potential as well as the immunophenotype of BMMSCs expanded in serum-free and serum-containing media. Among others, they tested the MC-XF medium and made the observation that serum-free media supported a higher proliferation capacity of BMMSCs maintaining their differentiation potential and immunomodulatory effects [26].

In the present study, we compared for the first time the JPC phenotype under animal-containing and animal-free culture conditions and made the interesting observation that the MC-XF culture medium promotes the growth and, most likely, the selection of the MSCA-1^+^ subpopulation out of the entire heterogeneous cell population derived from the jaw periosteum.

In a previous study from our laboratory, we were able to show that the MSCA-1^+^ cell fraction most likely represents the osteogenic progenitors within the entire population isolated from jaw periosteum. In addition to the fact that this fraction is a rare subpopulation (up to 5%), its magnetic separation results in poor cell survival/high mortality rates, indicating that only limited cell numbers could be available for prospective clinical applications using autologous cells. In vitro cell culturing in a xeno-free medium which would be able to select progenitor cells from a heterogeneous population could be a promising solution for successful tissue engineering approaches based on sufficient yields of stem cells.

The results obtained by life-monitoring measurements of unseparated JPCs revealed a significant decrease of cell impedance after the addition of the MC-XF culture medium due to the significant reduction of cell size. This observation was expected because of the absence of adhesion molecules normally contained in calf serum. However, the recovery of cell proliferation rates and, moreover, the cross-over of the proliferation curves obtained by DMEM-cultured cells indicate a much higher proliferative capacity of JPCs cultivated under xenogeneic-free culture conditions. We could confirm these results by the determination of PDT of unseparated JPCs of passage 4–7 growing under both culture conditions, as illustrated in table 1.

The here reported reduction in expression of the cell adhesion mediating surface markers CD29, CD90 and CD105 in MC-XF-cultured JPCs are consistent with those data published elsewhere based on comparing expression patterns of BMMSCs cultivated under FCS-containing and FCS-free conditions [27].

To clarify any doubts concerning the specificity of the von Kossa and Alizarin stains, we chose a more specific fluorescent staining of hydroxyapatite, the mechanism of which has not been revealed by the manufacturer. In general, by using OsteoImage, we detected an earlier mineralization capacity of MC-XF-cultured cells (fig. 5, upper panel). DMEM-cultured JPCs calcified at a later time point but to a higher extent compared to xeno-free-cultured cells (fig. 5, lower panel). However, as FCS is a complex natural product that may vary in the quality and concentrations of the contained proteins and growth factors from lot to lot and manufacturer to manufacturer, interpreting and comparing the obtained results are difficult. It is unknown whether nonspecific effects deriving from the contained FCS-components influence JPC mineralization.

Nodule formation emanates from almost every cell in the XF-cultivated monolayer. In contrast, the calcification potential of DMEM-cultured cells emanates only from a few cells of the entire cell population; these mineralization centers serve as aggregation points for further hydroxyapatite deposition. This observation indicates that cell cultivation using the MC-XF medium results in the emergence of a purer cell population, a fact that was confirmed by the flow cytometric analyses of unseparated JPCs for the determination of the percentages of MSCA-1^+^ cells (fig. 3) and the life-monitoring measurements of MSCA-1-separated JPCs (fig. 2). For both analysis approaches, cells were seeded first in DMEM to assess cell adherence. After gradual FCS reduction, MC-XF medium was added to the cells, resulting in the first step toward a significantly reduced cell size. The progression of proliferation curves indicates the preferential induction of MSCA-1^+^ cell fraction proliferation and a rather restrictive tendency with regard to the negative cell fraction. These results coincide with our experiences during cell cultivation in dishes/flasks coated with the attachment substrate (data not shown). We were not able to coat the E-plates for better cell adherence because signal transmission of the cell impedance was disturbed (data not shown). Therefore, we decided to conduct life-monitoring experiments with JPCs uniformly cultured in DMEM and to split them subsequently into DMEM- and MC-XF-cultured parallel test runs. As mentioned previously, the obtained results did not depend on the coating procedure. Furthermore, FACS analyses performed immediately after the conversion from DMEM to MC-XF cell cultivation clearly reveal a MSCA-1^+^ cell selection during the first 5–9 days, as summarized in table 2.

The obtained data from the gene expression analysis do not allow a precise statement due to the fact that JPCs growing under different conditions reach the different stages of osteogenesis at different time points. However, the significant higher induction of Runx-2 mRNA levels (at day 5 of osteogenesis) as well as of type I collagen (at day 10 of osteogenesis) and osteoprotegerin (at both analyzed time points) expression levels (fig. 7) in MC-XF- compared to DMEM-cultured JPCs, could reflect the observation of the earlier mineralization capacity observed in MC-XF-cultivated JPCs by OsteoImage.

Taken together, JPCs expanded in MC-XF proliferated faster, and nodule formation occurred earlier, originating from almost every cell of the monolayer; however, the degree of calcification seemed to be lower than in DMEM-cultivated cells. Interestingly, non-mineralizing JPCs showed mineralization capacity under MC-XF but not under DMEM culture conditions. Further studies should clarify whether JPC mineralization under MC-XF culture conditions is sufficient for successful tissue engineering applications. Therefore, analyses under three-dimensional culture conditions as well as *in vivo* in a suitable animal model are required. However, the clinical use of xeno-free MC-XF-cultured cells is limited due to the fact that this medium is still not in accordance with GMP-relevant qualifications.

Of even greater concern to us is the fact that JPC cultivation in the MC-XF medium leads to the preferential proliferation of the subpopulation with the highest osteogenic potential. This would prevent the stressful cell magnetic separation and the prolonged *in vitro* passaging of JPCs and thus speed up the *in vitro* culture procedures in this stem cell type.
